# Service-Chain-Driven Communication and Computing Integration Networking: A Case Study of Levee Piping Hazard Inspection via Remote Sensing

**DOI:** 10.3390/s25134187

**Published:** 2025-07-04

**Authors:** Jing Chen, Lyuzhou Gao, Hongquan Sun, Siquan Yang, Zhonggen Wang, Yuting Wan, Kedi Wang

**Affiliations:** 1 National Institute of Natural Hazards, Ministry of Emergency Management of the People’s Republic of China, Beijing 100085, China; chenjing@ninhm.ac.cn (J.C.); sunhq@ninhm.ac.cn (H.S.); siquanyang@ninhm.ac.cn (S.Y.); zhonggenwang@ninhm.ac.cn (Z.W.); 2 Key Laboratory of Compound and Chained Natural Hazards Dynamics, Beijing 100085, China; 3 State Key Laboratory of Information Engineering in Surveying, Mapping and Remote Sensing, Wuhan University, Wuhan 430079, China; wanyuting@whu.edu.cn; 4 School of Safety Science, Tsinghua University, Beijing 100084, China; codywang@mail.tsinghua.edu.cn

**Keywords:** integration networking architecture, collaborative computation, service chains, remote sensing application, hazard identification

## Abstract

Computing power network (CPN) is designed to utilize multi-dimensional resources to complete computing tasks. However, in practical applications, the CPN architecture has difficulty in coordinating cross-domain heterogeneous resources, making it impossible to achieve the real-time and high scalability requirements of computationally intensive and time-sensitive tasks such as levee piping hazard inspection via remote sensing in emergency scenarios. Based on this, we propose a communication and computation integrated network architecture, referred to as (Com)^2^INet, that integrates “sensing”, “transmission”, and “computation” phases. In the sensing phase, thermal infrared imagery is utilized to retrieve land surface temperature fields through radiative transfer mechanisms, providing a reliable foundation for visual segmentation of piping hazards. In the transmission phase, we adopt the designed multi-path transmission mechanism to promote the efficient data flow across heterogeneous networks. In the computation phase, the proposed SACM algorithm, which is functionally decomposed and implemented as service chains within the proposed network architecture, dynamically processes the retrieved temperature fields to achieve precise hazard identification. This integrated framework ensures seamless interaction between sensing, communication, and computation, addressing the challenges of real-time hazard detection in emergency scenarios.

## 1. Introduction

With the advent of the era of intelligent everything, various new application scenarios are constantly emerging, such as augmented reality, ultra-high-speed railway, holographic communication, and smart industrial Internet of Things [1]. A large number of heterogeneous terminal devices are connected to the Internet, generating tremendous data at the edge of the network, which needs to be efficiently transmitted and processed [2]. According to the Internet Data Center, by 2025, the annual data generated worldwide will grow to 175 Zettabytes [3]. By leveraging the massive amount of increased data, various advanced services with customized requirements can be provided to enhance the comprehensive analysis capacities of complex application scenarios. For instance, in the scenario of ultra-high-speed railway, the obtained audio and video data are used to effectively ensure the operation safety of ultra-high-speed railway vehicles through artificial intelligence (AI) algorithms. In the scenario of emergency response, the remote sensing data are used to improve rescue efficiency through AI algorithms. These services have the common characteristics of being compute-intensive and time-sensitive. Therefore, how to promote efficient computing is particularly critical to support plenty of advanced services in the intelligent everything scenarios.

New computing paradigms have been proposed to achieve the above target. Driven by powerful computing capabilities, cloud computing with large storage capacity and high data throughput has become the main platform for the application of AI algorithms [4]. However, this “in-network computing” paradigm has problems such as extended decision transmission delay and increased network burden when interacting with terminal devices. By sinking computing and storage resources from the cloud to edge of the network, edge computing paradigm and fog computing paradigm have arisen, reducing task transmission delay and greatly promoting the development of AI technology [5,6]. With limited computing resources, the compute-intensive tasks are offloaded to multiple edge nodes to collaboratively utilize decentralized computing resources, leading to data privacy issues. To this end, the distributed computing [7], such as federated learning, has attracted widespread attention from industry and academia. As advanced services tend to be customized, diversified, and intelligent, a single computing paradigm is difficult to meet the increasingly complex quality of service (QoS) and quality of experience (QoE) demands. Therefore, it is crucial to build a unified computing platform that integrates multiple computing paradigms, in which massive concurrent computing tasks can be globally orchestrated more efficiency to guarantee advanced service requirements.

Orchestrating massive concurrent computing services on a unified computing platform faces the following challenges. First, orchestrating computing tasks requires dynamic scheduling of multi-dimensional heterogeneous resources from the perspectives of communication and computing. Moreover, considering the upper-layer diversity advanced services and underlying differentiated networks, the computing power and network in the existing networks are relatively independent. In addition, the unified computing platform needs to manage massive concurrent computing tasks generated by various advanced services such as live image analytics of remote sensing and large-scale AI model training, which makes it more complicated to flexibly schedule multi-dimensional heterogeneous resources to handle massive concurrent computing services. Therefore, it is necessary to design a unified computing platform to support customized services by coordinating the scheduling of multi-dimensional heterogeneous resources from the perspective of both communication and computation. Then, diversified computing services are giving rise to masses of QoS and QoE requirements, such as deterministic delay, model accuracy, clarity level, privacy protection, and so forth [8]. Therefore, it is necessary to design an intelligent resource scheduling algorithm to adaptively schedule multi-dimensional heterogeneous resources on the unified computing platform.

On the one hand, a novel communication and computation integrated network architecture, referred to as (Com)^2^INet, is proposed for intelligent scheduling of multi-dimensional resources in heterogeneous environments. Service-oriented principles and intelligence facilitate the integration of a software-defined network (SDN) [9], programming protocol independent packet processor (P4) technology [10], and network function virtualization (NFV) [11] into the designed architecture, enabling flexible network management and reduced operating costs. To be specific, SDN decouples data plane and control plane, supporting centralized control and device configuration. The combination of SDN and P4 technology allows network administrators to customize reconfigure network functions of data plane devices for supporting new functions and protocols, thereby offloading some network functions to the data plane, such as in-band telemetry [12], load balancing [13], and re-routing [14], to achieve flexible network management. Moreover, with the support of SDN and NFV technology, computing services can implement traditional network functions in the form of virtual network functions (VNFs), and can migrate from dedicated hardware devices to general-purpose devices to reduce dependence on hardware devices. In conclusion, huge computing flows can be scheduled across domains of end-edge-cloud, space–air–ground, and multiple data centers, enabling the deep integration of “computing and network”. On the other hand, we present a case study of an intelligent scheduling scheme with dynamic coordination of multi-dimensional resources for massive remote sensing images, where a stepwise adaptive clustering approach is used to make the optimal joint decision for levee piping hazard identification.

Our main contributions are summarized below.

- The (Com)^2^INet integrates SDN, P4, and NFV technologies. It is a service-oriented computing paradigm that can schedule multi-dimensional heterogeneous resources across domains of end-edge-cloud, space–air–ground, and multiple data centers to ensure QoS and QoE requirements of advanced services. (Com)^2^INet architecture consists of three layers, i.e., a computing service layer, mapping adaption layer, and converged network layer, aiming to achieve the coordination of applications and network resources and the secure isolation of networks and applications.
- We design a service-centric computing service layer with different types of service identifications. The device-centric converged network layer with storage, forwarding and computing capabilities to process the service locally or forward it to other appropriate devices. The function-centric mapping adaption layer generates orchestration strategies to guide operations of converged network layer, vertically decouples computing service layer from converged network layer, and realizes the coordinated scheduling and intelligent integration of the service level and network level.
- With (Com)^2^INet as support and QoS requirements as constraints, we design a stepwise adaptive clustering method for levee piping hazard identification based on temperature field of UAV thermal infrared remote sensing imagery. The method utilizes a progressive approach to iteratively eliminate background regions and refine the target area, enabling the precise localization of piping hazards. Experimental results demonstrate that the proposed method outperforms benchmarks overall performance in terms of accuracy of target extraction and computational time of the algorithm.

The rest of the article is organized as follows. First, we present the (Com)^2^INet architecture in the Section 2. Following that, we present an application use case, in which the experimental results are given and discussed in the Section 3. Then, we elaborate on the directions and issues for future research in the Section 4. Finally, we conclude our work in the Section 5.

## 2. (Com)^2^INet: Communication and Computation Integrated Network Architecture

### 2.1. System Architecture

**Figure 1:**
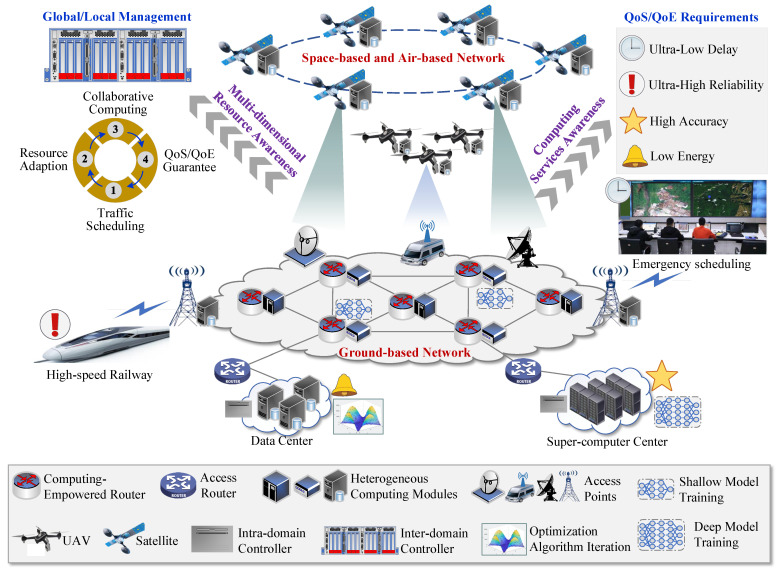
Illustration of the designed (Com)^2^INet.

As mentioned above, how to meet customized QoS and QoE requirements of advanced computing services remains a challenging problem [15]. To this end, a novel system architecture integrating multi-dimensional resources and heterogeneous networks, abbreviated as (Com)^2^INet, is proposed, as shown in Figure 1. The architecture aims to integrate ubiquitous connectivity and collaborative computation, jointly scheduling heterogeneous resources from both communication and computation perspectives to solve the dilemma that emerging applications require more computing power while available computing power is ubiquitous but not easy to use.

The (Com)^2^INet network has the following new features compared with the traditional forwarding dedicated networks. (1) Heterogeneous network components, such as router devices, end devices, edge servers, cloud servers, unmanned aerial vehicle (UAV) and satellites, etc., are equipped with computing units to handle massive concurrent computing tasks [16]. In addition, the designed architecture includes multiple computing centers such as data centers and supercomputer centers [17]. Therefore, massive concurrent flows can be scheduled across domains of end-edge-cloud, space–air–ground, and multiple data centers and support the deep integration of “computing and network” to efficiently satisfy complex QoS and QoE requirements of customized advanced computing services. (2) A hierarchical control mechanism is adopted to jointly perform global and local management. To be specific, the controllers are deployed in each domain to achieve dynamically adjust the network behaviors and manage multi-dimensional network resources. The master controller is used as a cross-domain orchestrator to centrally control the entire (Com)^2^INet. It can communicate with other controllers in each domain through the eastbound/westbound interface to exchange aggregated control information [18].

**Figure 2:**
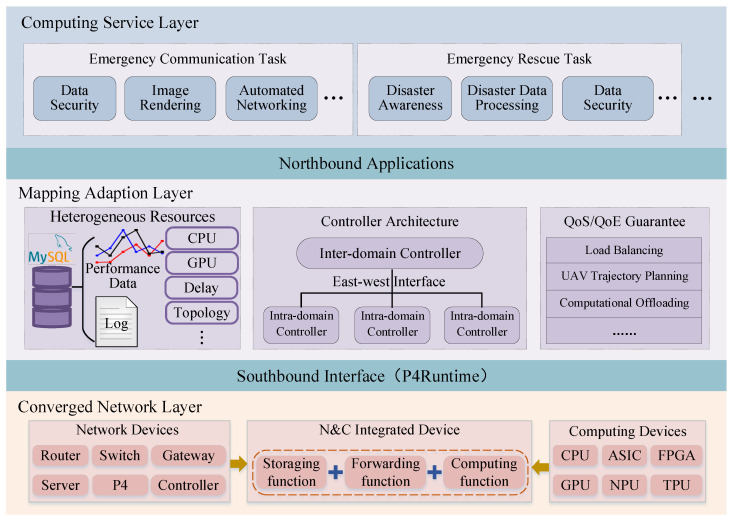
Functional architecture of the designed (Com)^2^INet.

The hierarchical control mechanism divides the functional architecture into three layers, in terms of computing service layer, mapping adaption layer, and converged network layer, as shown in Figure 2. We next describe each layer in the (Com)^2^INet functional architecture in detail below.

Computing Service Layer takes service as the basic unit. The computing power identifier (CID) is used to identify the computing services, and the computing power behavior description (CBD) is used to identify QoS and QoE requirements. To be specific, the attribute characteristics of customized computing services can be identified according to their QoS and QoE requirements to obtain useful information, such as whether the task needs to be split and offloaded to multiple computing devices for processing, how many computing devices need to be scheduled, and which optimal devices to schedule to complete the task. The above critical information can provide service-level support for the integrated scheduling of “computing and network” [19]. In addition, the computing tasks can be divided into different types, such as emergency communication tasks and emergency rescue tasks, and further divided into general computing tasks and intelligent computing tasks to facilitate global task management. Generally, computational process of general computing tasks with models that require global consistency or data integrity, such as linear regression [20] and K-means [21], should be executed on an edge server with the required mathematical tool installed and cannot be split and be performed on separate computing devices. Conversely, the computation process of intelligent computing tasks with AI models that require privacy protection or distributed computing, such as federated learning [22] and deep neural network [23], can be split and coordinated among multiple computing devices.

Converged Network Layer takes network component as the basic unit and is mainly responsible for data storage, forwarding, and computing, including network devices, computing devices, and integrated devices. The node identifier (NID) is used to identify the network component, and the node behavior description (NBD) is used to identify network functions. To be specific, network devices such as switches, routers, and gateways constitute the core network and are mainly responsible for data storage and forwarding. The computing devices are composed of one or more processing units such as central processing unit (CPU), network processing unit (NPU), field programmable gate array (FPGA), and graphics processing unit (GPU), which are responsible for processing real-time tasks [24]. Computing devices are distributed at the edge of the network, including multiple computing centers, compared with network devices. Integrated devices integrate computing, storage, and forwarding functions. For instance, router devices can have storage, forwarding, and computing functions by introducing the computing function. When the computing-enhanced router device receives a service, it can process the service locally or forward it to other appropriate devices. Note that network components support heterogeneous network protocol communications including IPv4, IPv6, etc., enabling a cross-protocol collaborative transmission mechanism.

Mapping Adaption Layer takes the function as the basic unit and is mainly responsible for scheduling multi-dimensional resources. The function identifier (FID) is used to identify the function types, and the function behavior description (NBD) is used to identify function functions. To be specific, there are three computing paradigms that support the implementation of computing services, in terms of in-network computing [25], out-of-network computing [26], and end-edge-cloud collaborative computing [27]. In-network computing can directly execute lightweight computing tasks in network devices, such as switches and routers, along the task transmission path, instead of transmitting data to traditional computing nodes, such as servers and cloud platforms. Out-of-network computing, with powerful computing capabilities, can orchestrate heavy-weight computing tasks to data centers and supercomputer centers for execution. Moreover, a computing task can be decomposed into multiple micro-services, or a monolithic function can be divided into multiple portions to orchestrate different parts between ends, edges, and clouds. For instance, the deep neural network (DNN) model can be partitioned, where the shallow layers are deployed and performed on the ends, the middle layers are deployed and performed on edges, and the other layers are deployed and performed on the cloud.

Between the computing service layer and the mapping adaption layer, a behavior matching mechanism is used. The dynamic matching process from computing services to functions is formulated as below.

(1) $$\left[\begin{array}{c} FID_{1}\\ FID_{2}\\ \vdots \\ FID_{s} \end{array}\right]≜\Omega \left(\left[CID_{i},CBD_{i}\right],\left[\begin{array}{c} FID_{1},FBD_{1}\\ FID_{2},FBD_{2}\\ \vdots \\ FID_{m},FBD_{m} \end{array}\right]\right),$$

where $\left[FID_{1},FID_{2},\cdots ,FID_{s}\right]$ is the group identifier of the *s* functions selected according to the requirements of on-demand networking. $\left[CID_{i},CBD_{i}\right]$ represents the *i*-th computing service, $\left[\left(FID_{1},FBD_{1}\right),\left(FID_{2},FBD_{2}\right),\cdots ,\left(FID_{m},FBD_{m}\right)\right]$ are *m* pairs of FID and FBD in the network, and Ω(·) is the behavior matching mapping function.

Between the mapping adaptation layer and the converged network layer, a behavior clustering mechanism is used. The intelligent clustering process from functions to network components is formulated as below.

(2) $$\left[\begin{array}{c} {\left\{NID_{11},NID_{12},\ldots ,NID_{1m_{1}}\right\}}_{CID_{1}}\\ {\left\{NID_{21},NID_{22},\ldots ,NID_{2m_{2}}\right\}}_{CID_{2}}\\ \vdots \\ {\left\{NID_{R1},NID_{R2},\ldots ,NID_{Rm_{R}}\right\}}_{CID_{R}} \end{array}\right]≜\Phi \left(\left[\begin{array}{c} FID_{1},FBD_{1}\\ FID_{2},FBD_{2}\\ \ldots \\ FID_{R},FBD_{R} \end{array}\right],\left[\begin{array}{c} NID_{1},NBD_{1}\\ NID_{2},NBD_{2}\\ \ldots \\ NID_{R},NBD_{R} \end{array}\right]\right)$$

where $\left\{NID_{i1},NID_{i2},\ldots ,NID_{im_{i}}\right\}$ represents the set of network components served by the *i*-th computing service, $NID_{ij}$ represents the *j*-th network component serving the *i*-th computing service, $m_{1}-m_{R}$ represents the number of network components served by the *R* computing services, and Φ(·) is the behavior clustering mapping function.

The core of this computing paradigm lies in establishing the relationships among computing services, functional groups, and network components. To achieve resource scheduling and service guarantees, the following steps are implemented. (1) Service-Group Mapping: based on customized task requirements and fine-grained heterogeneous resources from both communication and computation perspectives, functional modules such as inter-domain scheduling, intra-domain scheduling, and QoS/QoE guarantees are constructed. These modules match the most optimal functions to different computing services, enabling dynamic “service-group” mapping. (2) Group-Component Mapping: leveraging the obtained [FID,FBD] and [NID,NBD] information, heterogeneous resource scheduling strategies are generated. The corresponding network components are then clustered to execute network configuration files issued by the mapping adaption layer. This process completes service/network-level configuration and function deployment, thereby realizing “group-component” mapping and ensuring the execution of computing services.

(Com)^2^INet architecture decouples computing service layer from converged network layer vertically through the mapping adaptation layer, realizing the coordinated scheduling and intelligent integration of the service level and network level. On the one hand, (Com)^2^INet architecture is conducive to network providing users with customizable computing services, and on the other hand, it is also conducive to providing users with virtual private networks to satisfy the requirements of related computing services in terms of reliability, security, and scalability. Furthermore, (Com)^2^INet architecture horizontally decouples the control plane and the operation plane. The upper application plane communicates with the control plane through the northbound interface, and the underlying data plane communicates with the control plane through the southbound interface (e.g., P4Runtime). The control plane uniformly schedules heterogeneous network resources, translates decisions into configuration files, and sends them to the data plane to complete computing services.

### 2.2. Key Features of (Com)^2^INet Computational Paradigm

*Service-centric paradigm*. In the (Com)^2^INet computational paradigm, services become the core components and driving force of the network. The traditional network architecture is centered on data transmission. In (Com)^2^INet architecture, communication and computation are deeply integrated, and services need determine the allocation and scheduling of multi-dimensional resources. With the help of NFV technology, computing services are translated into corresponding service chains (SCs), and end-to-end services are realized by sequentially connecting the required network functions to satisfy the diverse requirements of communication and computation [28].

*Serverless-based model*. Network functions can be chained in an event-driven manner, typically initialized within milliseconds [29]. This on-demand chaining model enables the system to orchestrate different locations and differentiated resources in real time to achieve the optimal combination of user experience, resource utilization, and network efficiency. In addition, the model can easily update and replace specific network functions to adapt to new services or improve algorithm performance, enabling an efficient, intelligent, and elastic management paradigm.

*Regionless-based model*. In (Com)^2^INet architecture, heterogeneous network components are identified by names of network functions instead of IP addresses. (Com)^2^INet jointly considers heterogeneous resources from both the communication and computation perspectives, and schedules customized services to target network components for computing and analysis along optimal paths in a decentralized manner, achieving collaborative scheduling within the WAN. Regionless-based model solves the “IP bottleneck” problem and enables the application of new network architecture and service-centric paradigm [30].

In this paper, we propose a new computing paradigm, named (Com)^2^INet, which jointly schedules heterogeneous resources from both communication and computation perspectives to solve the dilemma that emerging applications require more computing power while available computing power is ubiquitous but not easy to use.

### 2.3. Key Technologies

**Figure 3:**
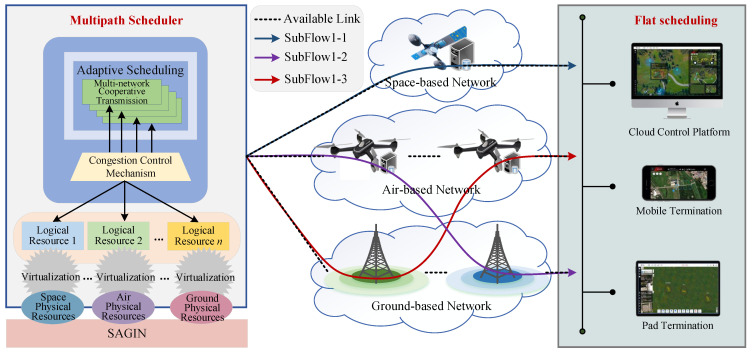
Illustration of multi-path transmission.

(1) *Measurement and modeling of multi-dimensional heterogeneous resources*. The computing capability of network component *n*, referred to as $CC_{n}$, is measured by indicators such as FLOPS, IOPS, BOPS, LOPS, etc., so as to satisfy the differentiated calculation rate requirements of complex and diversified services. These are mathematical expressed as $CC_{n}=\left\{FLOPS_{n},IOPS_{n},BOPS_{n},LOPS_{n},\cdots \right\}$. The storing capability of network component *n*, referred to as $SC_{n}$, is measured by indicators such as disk capacity DC, disk bandwidth DB, Input/Output Per Second IOPS, memory capacity MC, memory bandwidth MB, etc. These are mathematical expressed as $SC_{n}=\left\{DC_{n},DB_{n},IOPS_{n},MC_{n},MB_{n},\cdots \right\}$. The networking capability of network link *l* is measured by indicators such as bandwidth BW, end-to-end delay *D*, delay jitter DJ, packet loss rate PL, throughput TP, etc. These are mathematical expressed as $NC_{l}=\left\{BW_{l},D_{l},DJ_{l},PL_{l},TP_{l},\cdots \right\}$.

(2) *Multi-path transmission*. In the (Com)^2^INet computational paradigm, multi-path transmission mechanism has the characteristics of dynamic awareness, resource coordination, and efficient utilization, and supports data parallelism and task splitting, as described in Figure 3. By perceiving task requirements, network environment, and distribution of multi-dimensional resources, and by comprehensively considering factors such as network load, traffic type, and delay constraints, it intelligently selects multiple optimal paths to achieve cross-regional, multi-link adaptive transmission. This mechanism can avoid congestion on a single path, improve data transmission efficiency, and improve multi-dimensional resource utilization.

(3) *SC-based heterogeneous resource scheduling*. In the (Com)^2^INet architecture, SC-based heterogeneous resource scheduling supports scheduling of massive concurrent computing tasks across multiple computing domains such as end-edge-cloud, space–air–ground, and multiple data centers. To be specific, computing services can be modeled as multi-objective joint optimization with multi-network constraints and translated into corresponding SCs, which are composed of atomic network functions. SCs can be deployed and performed between ends, edges, and clouds, scheduling resources in heterogeneous infrastructure pools to satisfy the diverse requirements of communication and computation. It is worth noting that two or more domains can be selected simultaneously to effectively coordinate computing, storage, and network resources with different geographical distributions to provide computing services for tasks with extremely strict QoS and QoE requirements. Heterogeneous resource scheduling requires comprehensive consideration of task characteristics (i.e., parallel mode of large model training), traffic patterns (i.e., mixed long and short flows, burst flows), network topology, and other factors, and achieves global optimal resource adaptation and computing task execution efficiency by building an efficient, flexible, and intelligent adaptive optimization form.

(4) *Fault tolerance*. In the (Com)^2^INet architecture, a fault-tolerance mechanism is designed to ensure that in a complex network environment, when faced with the network component/link failure, overload, or other sudden abnormal situations, the abnormal point can be quickly located through the intelligent fault detection and diagnosis system. The mechanism comprehensively considers multi-dimensional factors such as network topology, resource utilization, and QoS and QoE requirements, and based on preset fault tolerance strategies and real-time network status, dynamically performs operations such as intelligent re-routing, dynamic load balancing, or flexible migration of network functions to ensure the continuity of data transmission, uninterrupted critical services, and undegraded service quality.

### 2.4. Working Principle

**Figure 4:**
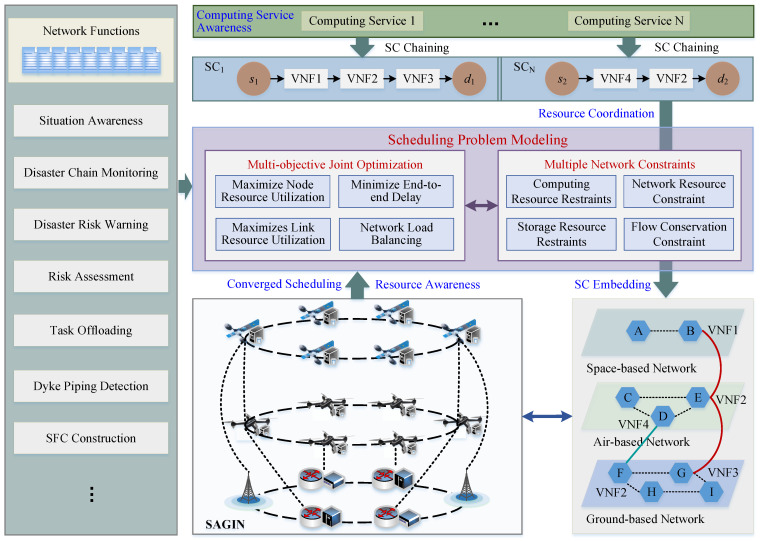
Working principle for implementing computing services in the (Com)^2^INet architecture.

The working principle for implementing computing services in (Com)^2^INet architecture is depicted in Figure 4. The environment awareness module perceives computing service requirements, network functions, and multi-dimensional heterogeneous resources from both the communication and computation perspectives. To be specific, computing services measurement translates computing service requirements into actually required heterogeneous resources and customized network functions through intent translation to improve the efficiency of interaction with users. The network function measurement is to measure the heterogeneous resources required for different functions, so as to effectively understand the heterogeneous resources required for computing services call functions. The perceived translated services and heterogeneous resources are notified to scheduling problem modeling serverless platform for modeling multi-objective joint optimization with multi-network constraints. Customized network functions are chained, executed, and billed according to current services. Specifically, a serverless platform builds SCs for arrival services, and SCs schedule resources in the heterogeneous infrastructure pooling.

During orchestrating phase, we establish scheduling mechanism from three perspectives: users, networks, and providers. First, the scheduling mechanism should adaptively satisfy various QoS requirements from a user’s perspective, in terms of delay requirement, heterogeneous resources requirement, ultra-reliable transmission, and so forth. Then, the (Com)^2^INet infrastructure integrates awareness, forwarding, storage, computing, and processing capabilities, which puts forward greater flexibility requirements for network management. Moreover, an effective resource scheduling mechanism should enable the provider to obtain the maximum benefit to motivate more providers to participate in the communication and computation integrated (Com)^2^INet. Furthermore, the obtained scheduling mechanism is translated into the network configuration files, which guide the optimal network components to host required SCs to accomplish the computing service in serial or parallel manner.

The (Com)^2^INet architecture introduces an identifier resolution system (e.g., CID, FID, NID) to integrate computing resources into a unified scheduling framework, enabling coordinated control and optimization of computing, networking and storage resources. This promotes a shift from “connection-centric” to “service-centric”, providing computing power as a service. Furthermore, (Com)^2^INet architecture implements atomic-level multi-dimensional resource collaborative scheduling through unified identifiers, rather than limiting scheduling granularity to the virtual machine or container level, thereby eliminating service delays and resource waste. In summary, the (Com)^2^INet architecture can realize the mapping from computing service to a set of network components through the intelligent integration of multi-space and multi-dimensional heterogeneous resources, so as to facilitate dynamic and on-demand adjustment of services and networks.

### 2.5. Implementing Computing Services on the (Com)^2^INet Platform

In practical system applications, the observations phase is implemented through a dual monitoring approach. The system state is jointly monitored by a Network Observer module installed inside the centralized SDN controller through northbound API and an in-band network telemetry (INT) collector. For instance, multi-dimensional resource measurement quantifies computing capabilities (e.g., FLOPS, IOPS), storage metrics (e.g., disk capacity, bandwidth), and network parameters (bandwidth, delay), forming a standardized model for comprehensive resource characterization. Meanwhile, users submit computing tasks through the (Com)^2^INet platform. The platform’s service orchestration module analyzes each task’s resource requirements, performance objectives, geographical constraints, and other conditions, converting them into executable data flows. Upon fetching observations, pretrained models, such as the multi-path transmission model, heterogeneous resource scheduling, and fault tolerance, make optimal decisions for the computing tasks. The orchestration policies are delivered to the orchestrator, while routing policies are delivered to the controller, ensuring that the management module and infrastructure are fully decoupled. Network configuration will affect network performance, which would in turn be reflected in their reported performance metrics. Based on the reported performance metrics, the policy model can dynamically adjust network policies to adapt to fluctuating traffic patterns and unpredictability network. To sum up, the (Com)^2^INet platform facilitates a closed-loop “measure-adapt-optimize” process that ensures optimal computing service delivery.

## 3. Use Case: (Com)^2^INet for Levee Piping Hazard Inspection via Remote Sensing

Piping leakage is a critical factor leading to levee breaches and catastrophic flooding disasters. Levee breaches often result in mass casualties, making piping leakage a major threat to flood disaster prevention and mitigation in China. According to statistics, China’s river levees extend over 328,000 km, protecting a population of 650 million and 42 million hectares of farmland. However, more than 90% of these river levees consist of aging earth-rock dams, which are highly susceptible to severe hazards during the annual flood season due to high water levels. UAV-based remote sensing technology has demonstrated significant potential in the inspection and identification of piping hazards in levees. UAV platforms are characterized by their portability, lightweight design, and low cost, enabling rapid acquisition of levee data. Furthermore, various sensors can capture diverse morphological data of levees, providing abundant information sources for identifying piping hazards. The remote sensing data processing techniques designed based on the (Com)^2^INet architecture can achieve rapid, accurate, and large-scale inspections of levee hazards.

However, current UAV-based remote sensing approaches for levee piping hazard inspection face challenges such as low recognition accuracy and poor scalability. Although piping hazards are visually prominent in thermal infrared imagery, such imagery is heavily influenced by weather conditions. The piping features extracted from thermal infrared imagery exhibit significant variability under different weather and environmental conditions, leading to poor stability in distinguishing piping hazards from other surface objects. Moreover, the complex flood-season levee environment introduces interference to recognition algorithms. The levee surface is covered by diverse natural and human-made objects, and the harsh conditions during the flood season further complicate the scene. Particularly in large-scale, multi-sensor, and multi-object application scenarios, uncertain objects often affect the performance of recognition algorithms.

Currently, deep-learning-based object recognition algorithms typically rely on large sample datasets. However, due to the difficulty in obtaining piping hazard data, the demand for extensive training samples cannot be met. This study proposes an unsupervised machine-learning-based method, the SC-based stepwise adaptive clustering method (SACM), for piping hazard segmentation using UAV thermal infrared imagery. The SC-based SACM method extracts piping hazards through iterative clustering and adaptive strategies. First, thermal infrared imagery is converted into temperature maps. Second, a series of binary classifications is applied to the temperature maps to progressively exclude background regions unrelated to piping hazards, thereby mitigating interference caused by complex levee environments. Finally, the elbow method is introduced to determine the hyperparameters of the clustering algorithm, overcoming the dependence on subjective manual settings for cluster centers and achieving adaptive clustering based on data features.

**Figure 5:**
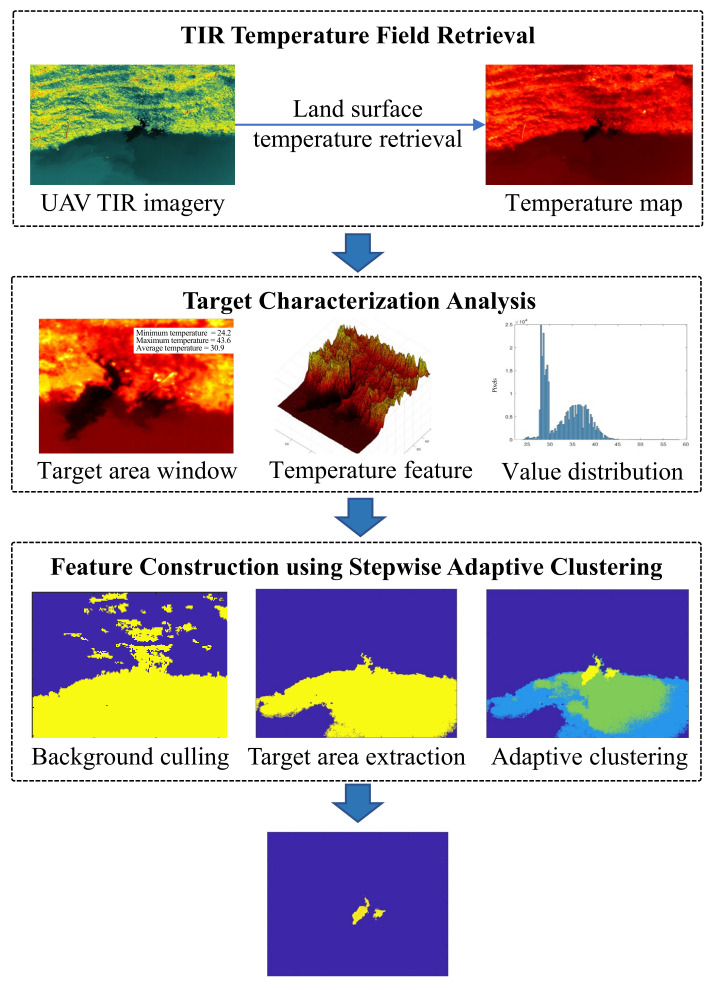
Illustration of SC-based SACM for levee piping hazard inspection via remote sensing.

SC-based SACM is an unsupervised clustering algorithm specifically designed for piping hazard segmentation in UAV thermal infrared imagery. Unlike traditional clustering algorithms, SC-based SACM employs a progressive and adaptive strategy to iteratively refine the segmentation process. By leveraging temperature field analysis and stepwise clustering, SC-based SACM effectively eliminates irrelevant background regions and accurately extracts piping hazard targets. This method is particularly robust in complex environments, ensuring high precision and reliability in hazard detection. The SC-based SACM algorithm is composed of three key steps: temperature field preprocessing, background removal, and stepwise adaptive clustering. Each step can be modeled as a VNF and chained into an SC, which can be executed sequentially on multiple computing devices to achieve the scalable levee piping hazard inspection. The SC-based SACM algorithm is designed to overcome challenges such as interference from complex levee environments, variability in thermal infrared imagery due to weather conditions, and the subjectivity of manual parameter selection. By introducing the elbow method for hyperparameter determination, the SC-based SACM achieves fully adaptive clustering based on data characteristics, without requiring predefined cluster centers. The specific process is illustrated as Figure 5, and the main pseudo-code is as shown as Algorithm 1.
**Algorithm 1** SC-based SACM for levee piping hazard inspection.**Input**: UAV thermal infrared imagery *I*; Convergence threshold ϵ; Maximum iterations *T*. **Process**:
1. **Temperature Field Preparation**:
· Convert the thermal infrared imagery *I* into a temperature map *T* using the thermal infrared radiative transfer mechanism.
· Normalize the temperature map *T* to ensure consistent data scaling across different images.
2. **Background Removal**:
· Perform a series of binary segmentations on the temperature map *T* to isolate potential piping hazard regions:
· Calculate the temperature threshold τ for binary segmentation using statistical analysis of the temperature distribution.
· Segment the image into background regions and suspected target regions based on τ.
· Iteratively refine the segmentation by updating τ to progressively exclude irrelevant background regions.
3. **Stepwise Adaptive Clustering**:
· Apply adaptive clustering to the refined target regions:
· Use the elbow method to determine the optimal number of clusters *k* based on the within-cluster sum of squares (WCSS)
· Initialize cluster centroids adaptively based on the temperature distribution of the target regions.
· Perform iterative clustering to assign each data point to the nearest cluster and update cluster centroids.
· Continue the clustering to assign each data point to the nearest cluster and update cluster centroids.
4. **Output**:
· Extracted piping hazard regions with precise boundaries.
· Cluster assignments $U=\{u_{1},u_{2},\ldots ,u_{k}\}$ representing the segmented target areas.


(i) *Land surface temperature retrieval from UAV thermal infrared imagery*. Utilizing thermal infrared (TIR) imagery acquired by UAVs, the land surface temperature is retrieved according to the thermal infrared radiative transfer mechanism. Specifically, for UAV-based single-band TIR imagery, the land surface temperature $T_{s}$ is estimated using the radiative transfer equation.

(3) $$L_{\lambda }=\varepsilon_{\lambda }B_{\lambda }\left(T_{s}\right)\tau_{\lambda }+\left(1-\varepsilon_{\lambda }\right)L_{\lambda }^{↓}\tau_{\lambda }+L_{\lambda }^{↑},$$

where $L_{\lambda }$ is the at-sensor radiance, $\varepsilon_{\lambda }$ is the surface emissivity (set to 0.95), $B_{\lambda }\left(T_{s}\right)$ is the blackbody radiance at surface temperature $T_{s}$, $\tau_{\lambda }$ is the atmospheric transmittance, $L_{\lambda }^{↑}$ is the upwelling atmospheric radiance, and $L_{\lambda }^{↓}$ is the downwelling atmospheric radiance. The parameters are set based on sensor calibration and local atmospheric conditions.

After inversion, the resulting temperature map T is normalized to the range [0, 1] using the following formula.

(4) $$T_{norm}=\frac{T-T_{min}}{T_{max}-T_{min}},$$

where $T_{min}$ and $T_{max}$ are the minimum and maximum pixel values in the temperature map, respectively. This normalization ensures consistent data scaling across different images and provides a solid foundation for subsequent analysis.

(ii) *Levee piping temperature field analysis for image background removal*. Based on the retrieved temperature map, the thermal characteristics of piping targets are analyzed. To separate the image into background and suspected target regions, a statistical thresholding method is employed. The initial segmentation threshold is calculated as below.

(5) $$\tau =\mu_{T}+k\cdot \rho_{T},$$

where $\mu_{T}$ is the mean temperature, $\rho_{T}$ is the standard deviation of the temperature distribution, and *k* is an empirical parameter (typically set to 1.5 based on experiments). Binary segmentation is performed using this threshold to isolate potential piping hazard regions. The process is iteratively refined: after each segmentation, the threshold τ is updated according to the statistics of the remaining suspected target region. The iteration continues until the difference between the mean temperatures of the background and target regions falls below a predefined threshold (e.g., 1.5 K). This step effectively removes irrelevant background areas, reducing interference for subsequent clustering analysis.

(iii) *Feature construction using stepwise adaptive clustering*. Following background removal, stepwise adaptive clustering is employed to construct the thermal field features of the piping targets. The number of clusters *k* for the suspected target region is determined by the elbow method, which calculates the within-cluster sum of squares (WCSS) for a range of *k* values and selects the point where the decrease in WCSS becomes marginal, indicating the optimal cluster number. Initial cluster centroids are selected based on the peaks of the temperature histogram within the target region to enhance clustering stability. This process further refines the segmented target regions to extract suspected piping areas, avoiding the subjectivity associated with manual threshold selection. The SC-based SACM approach for levee piping hazard inspection is particularly robust and adaptable for extracting piping targets in complex scenarios, ensuring high scalability and accuracy in feature construction.

**Benchmarks**. (1) *k-Medoids* (KM) algorithm is a classical partitioning clustering algorithm widely used in data mining. Unlike k-Means, which relies on centroids as cluster representatives, KM selects actual data points (medoids) to represent clusters, making it more robust to noise and outliers. The algorithm aims to minimize the sum of dissimilarities between data points and their corresponding medoids, ensuring the formation of compact and well-separated clusters. The KM algorithm is particularly suitable for scenarios with non-Euclidean distance metrics or when the dataset contains outliers that could significantly distort the results of k-Means. By iteratively refining the medoids and cluster assignments, KM seeks to achieve optimal clustering with respect to the chosen dissimilarity measure. Because of the global dependence on the central object, the KM cannot be split and can only be executed as a whole function on a separate computing device. The main pseudo-code is as shown as Algorithm 2.

(2) *Fuzzy C-Means* (FCM) is a widely used unsupervised clustering algorithm based on fuzzy set theory. Unlike traditional hard clustering methods, such as k-Means, where each data point is assigned to exactly one cluster, FCM allows a data point to belong to multiple clusters with varying degrees of membership. This flexibility makes FCM particularly suitable for applications where data points exhibit overlapping characteristics or uncertainty in cluster boundaries. The objective of FCM is to minimize the weighted sum of squared errors between data points and cluster centers, with the weights determined by the membership degrees. The algorithm iteratively updates cluster centers and membership degrees until convergence. Since FCM requires global consistency, it cannot be split and can only be executed as a whole function on a separate computing device. The main pseudo-code is shown as Algorithm 3.
**Algorithm 2** KM-based levee piping hazard inspection.**Input**: Dataset $D=\{x_{1},x_{2},\ldots ,x_{m}\}$; Number of clusters *k*. **Process**:
1. Randomly select *k* samples from *D* as the initial centroids $\{\mu_{1},\mu_{2},\ldots ,\mu_{k}$}.
2. **Repeat**: 3. Initialize $C_{i}=\emptyset$ for 1≤i≤k. 4. For j=1,2,…,m:
· Compute the distance between sample $x_{j}$ and each centroid $\mu_{i}\left(1\leq i\leq k\right):d_{ji}=\left|\right|x_{j}-\mu_{i}{\left|\right|}_{2}$.
· Assign $x_{j}$ to the cluster with the nearest centroid: $\lambda_{j}=argmin_{i\in \{1,2,\ldots ,k\}}d_{ji}$.
·Update the cluster: $U_{\lambda_{j}}=U_{\lambda_{j}}\cup \left\{x_{j}\right\}$.
v. **End For**
vi. **For** i=1,2,…,k:
· Compute the new centroid for each cluster: $\mu_{i}^{\prime }=\frac{1}{U_{i}}\sum_{x\in U_{i}}x$.
**If**
$\mu_{i}^{\prime }\neq \mu_{i}$:
· Update the centroid: $\mu_{i}=\mu_{i}^{\prime }$.
**Else**:
· Keep the current centroid unchanged.
vii. **End For**
3. **Until** centroids remain unchanged or maximum iterations are reached.
**Output**: Cluster assignments $U=\{u_{1},u_{2},\ldots ,u_{k}\}$.


**Algorithm 3** FCM-based levee piping hazard inspection.**Input**: Dataset $D=\{x_{1},x_{2},\ldots ,x_{m}\}$; Number of clusters *k*; Fuzziness parameter m>1; convergence threshold ϵ>0; Maximum iterations *T*. **Process**:
1. **Initialization**:
· Randomly initialize the membership matrix $H=[h_{ij}]$, where $h_{ij}\in \left[0,1\right]$ and $\sum_{i=1}^{k}h_{ij}=1$ for all *j*.
2. **Repeat**: 3. **Update Cluster Centers**:
· Compute the cluster center $c_{i}$ for $i=1,2,\ldots ,k:c_{i}=\frac{\sum_{j=1}^{m}h_{ij}x_{j}}{\sum_{j=1}^{m}h_{ij}}$.
iv. Update Membership Degrees:
· For each data point $x_{j}$ and cluster *i*, update the membership degree $h_{ij}$:
$h_{ij}=\frac{1}{\sum_{l=1}^{k}{\left(\frac{\left|\right|x_{j}-c_{i}\left|\right|}{\left|\right|x_{j}-c_{l}\left|\right|}\right)}^{\frac{2}{m-1}}}$.
v. Check Convergence:
· Compute the change in membership matrix δH. If ▵H<ϵ or the maximum nember of iterations reached, stop the iteration.
3. **End Repeat**.
4. Assign each data point $x_{j}$ to the cluster with the highest membership degree.
**Output**: Cluster centers $U=\{u_{1},u_{2},\ldots ,u_{k}\}$; Membership matrix $H=[h_{ij}]$.


(3) *Gaussian mixture model* (GMM) is a probabilistic model widely used for clustering tasks. It assumes that data points are generated from a mixture of several Gaussian distributions with unknown parameters. Each Gaussian component represents a cluster, and the GMM algorithm aims to model the entire dataset as a weighted sum of these Gaussian distributions. GMM is particularly effective for datasets with overlapping clusters and can model more complex cluster shapes compared to simpler algorithms like k-Means. Unlike hard clustering methods, such as k-Means, which assign each data point to a single cluster, GMM performs soft clustering by assigning each data point a probability of belonging to each cluster. This probabilistic approach makes GMM more flexible in capturing underlying structure of the data. For the soft clustering process of GMM, the posterior probability calculation and parameter update must be based on global data to ensure model convergence and accuracy. Therefore, the GMM model cannot be split and can only be executed as a whole function on a separate computing device. The main pseudo-code is shown as Algorithm 4.
**Algorithm 4** GMM-based levee piping hazard inspection.**Input**: Dataset $D=\{x_{1},x_{2},\ldots ,x_{m}\}$; Number of clusters *k*; convergence threshold ϵ>0. **Process**:
1. **Initialization**:
· Randomly initialize the parameters of the Gaussian components.
· Means $\chi_{i}\left(i=1,\ldots ,k\right)$.
· Covariance matrices $\sum_{i}\left(i=1,\ldots ,k\right)$.
· Mixing coefficients π such that $\sum_{i=1}^{k}\pi =1$.
2. **Expectation-Maximization (EM) Algorithm**:
· **Repeat**:
· **E-step (Expectation)**:
· For each date point $x_{j}\left(j=1,\ldots ,m\right)$ and each Gaussian component i(i=1,…,k), compute the posterior probability (responsibility) that $x_{j}$ belongs to the i−th Gaussian: $\gamma_{ji}=\frac{\pi_{i}\cdot N\left(x_{j}|\chi_{i},\sum_{i}\right)}{\sum_{l=1}^{k}\cdot N\left(x_{j}|\chi_{l},\sum_{l}\right)}$ where $N(x_{j}|\chi_{i},\sum_{i})$ is the Gaussian probability density function: $N\left(x_{j}|\chi_{i},\sum_{i}\right)=\frac{1}{{\left(2\pi \right)}^{d/2}{\left|\sum_{i}\right|}^{1/2}}$.
· **M-step (Maximization)**:
· Update the parameters of the Gaussian components based on the responsibility $\gamma_{ji}$:
a. Update the mixing coefficients: $\pi_{i}=\frac{1}{m}\sum_{j=1}^{m}\gamma_{ji}$
b. Update the means: $\chi_{i}=\frac{\sum_{j=1}^{m}\gamma_{ji}x_{j}}{\sum_{j=1}^{m}\gamma_{ji}}$
c. Update the covariance matrices: $\sum_{i}=\frac{\sum_{j=1}^{m}\gamma_{ji}\left(x_{j}-\chi_{i}\right){\left(x_{j}-\chi_{i}\right)}^{T}}{\sum_{j=1}^{m}\gamma_{ji}}$
· **Check for Convergence**:
· Compute the log-likelihood of the data under the current model: $logL=\sum_{j=1}^{m}log\left(\sum_{i=1}^{k}\pi_{i}\cdot N\left(x_{j}|\chi_{i},\sum_{i}\right)\right)$.
· If the change in log-likelihood is less than the threshold ϵ stop the iteration.
3. **Output**:
· The parameters of the Gaussian components $\left(\chi_{i},\sum_{i},\pi_{i}\right)$ and the cluster assignments based on the maximum posterior probability: Cluster assignment for $x_{j}=argmax_{i}\gamma_{ji}$.


**Performance Evaluation**. *Evaluation Indicator*. Mean Intersection over Union (mIoU) and total runtime of per frame are adopted in this paper for evaluating accuracy of target extraction and algorithm efficiency. The mIoU quantitatively measures the accuracy of predicted segmentation results compared to the label truth. It evaluates the overlap between the predicted segmentation region and the true region for each class and computes the average across all classes. As a robust and interpretable metric, mIoU is particularly suitable for assessing the performance of clustering and segmentation algorithms, including those applied to UAV thermal infrared imagery for levee piping hazard detection. For the specific target, the IoU and the total runtime are computed as below.

(6) $$IoU=\frac{True\;Positive}{True\;Positive+False\;Positive+False\;Negative},$$

(7) $$T_{total}=T_{end}-T_{start},$$

where *True Positive* is the number of pixels correctly predicted as belonging to piping target, *False Positive* is the number of pixels incorrectly predicted as belonging to piping target, and *False Negative* is the number of pixels belonging to piping target.

**Figure 6:**
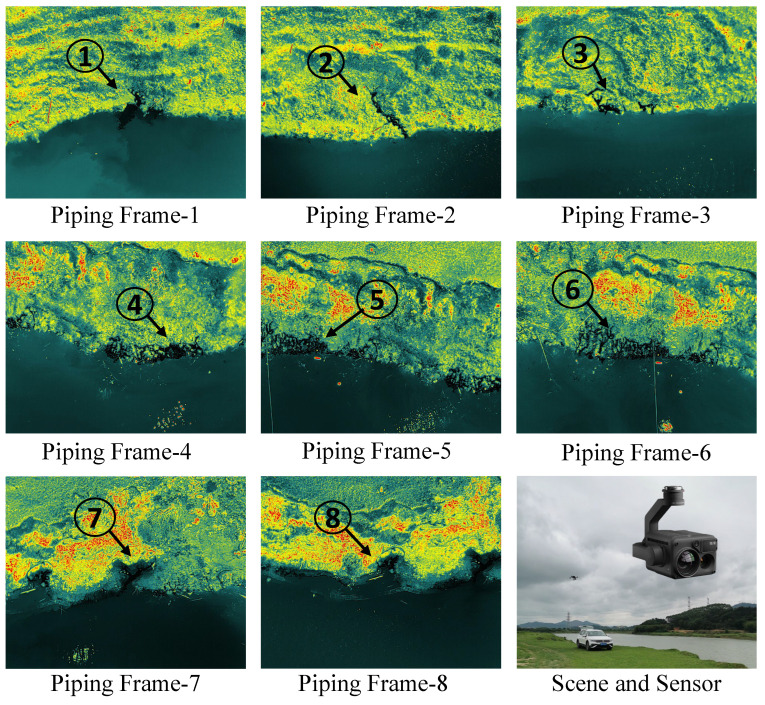
Eight frames of thermal infrared imagery with piping targets.

*Experimental Data*. The experimental data in this manuscript were obtained using remote sensing images captured by the DJI H20T sensor mounted on the DJI M300 UAV. The images were taken from a nadir perspective with a flight speed of 8 m per second, primarily acquiring thermal infrared remote sensing images. The data collection took place in the Pajiang River Basin, Fogang County, Qingyuan City, Guangdong Province, on 20 April 2023. The UAV operated at a flight altitude of 30 m, and the thermal infrared camera had a resolution of 640×512 with a Noise Equivalent Temperature Difference (NEDT) ≤50mK@f/1.0. During this data acquisition, eight frames of thermal infrared remote sensing images containing piping hazards were collected, as shown in Figure 6. These thermal infrared remote sensing images with piping hazards were used in this manuscript to test and evaluate the performance of the proposed SC-based SACM algorithm, in terms of the accuracy of target extraction and computational time of benchmarks.

**Figure 7:**
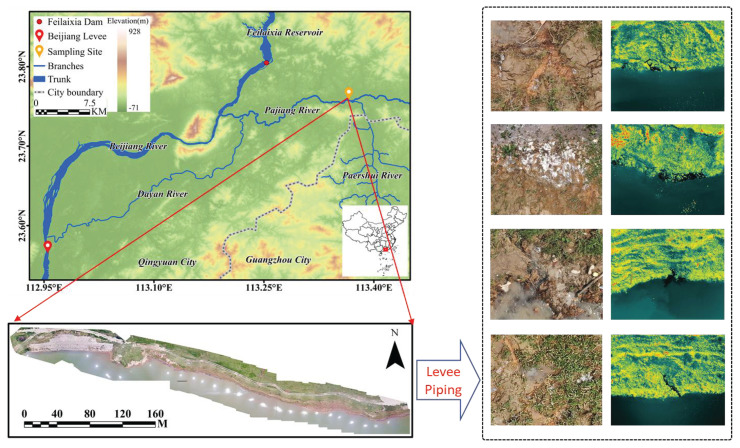
Study area and levee pipings.

To be specific, the study site is located in Qingyuan city, Guangdong province, China. Qingyuan city is characterized by complex terrain with undulating mountains. It has a subtropical monsoon climate, where summers are hot and winters are warm. The flood season occurs from April to September. During this period, precipitation is heavily influenced by monsoons and typhoons, making the area prone to flooding disasters. Moreover, the data was collected from the levee at the confluence of the Pajiang River and the Pa’ershui River. Due to its geographical location, this area is frequently affected by floods. As shown in Figure 7, the Pajiang River is a tributary of the Beijiang River. Its upstream area contains the largest comprehensive water conservancy project in Guangdong Province—the Feilai Gorge Reservoir. Downstream, there is the critical protective barrier for the Guangzhou metropolitan area—the Beijiang Levee. During the flood season, on one hand, the release of floodwater from the upstream reservoir causes the water level of the Pajiang River to rise significantly due to the backwater effect of the Beijiang River’s floodwaters, posing a threat to the safety of the levees. On the other hand, in order to protect the downstream levee, the Pajiang River basin has to be used as a flood storage and detention area. Therefore, the safety of the levee here not only affects both banks of the Pajiang River but may also impact the safety of the Guangzhou metropolitan area.

*Comparison in accuracy of target extraction*. As shown in Table 1, SACM demonstrates a significant advantage in terms of target extraction accuracy. As indicated by the data of Table 1, SACM consistently achieves the highest mIoU values across all tests, with an average mIoU of 0.1469. This suggests that SACM provides superior precision in extracting piping hazards. In contrast, traditional algorithms such as KM, GMM, and FCM exhibit lower average mIoU values of 0.0423, 0.0269, and 0.0303, respectively. These conventional methods often struggle with the complexities of thermal infrared remote sensing imagery, where background noise and environmental variations can adversely affect target extraction accuracy. SACM effectively mitigates irrelevant background regions through its progressive adaptive clustering strategy, enhancing the precision of piping hazard extraction. Its robust and reliable performance in complex environments is evident, maintaining stability under varying weather conditions. Consequently, SACM demonstrates superior performance in piping detection tasks, validating its applicability and efficacy in challenging scenarios.

*Comparison in computational time*. As shown in Table 2, SC-based SACM also exhibits a remarkable advantage in computational efficiency. SC-based SACM achieves the lowest average runtime per frame at 0.2288 s, significantly outperforming other algorithms. This indicates that SC-based SACM is highly efficient in processing thermal infrared imagery for piping hazard detection. In contrast, traditional monolithic-based algorithms, such as KM, GMM, and FCM, have average runtimes of 6.7628, 96.3845, and 209.3182 s per frame, respectively. To be specific, the time complexity of monolithic-based KM is $O\left(k{\left(n-k\right)}^{2}\right)$, where *n* indicates the number of data points and *k* indicates the number of clusters. The time complexity of monolithic-based GMM is $O(nkD^{2})$, where *D* indicates the dimension of the covariance matrix. The time complexity of monolithic-based FCM is O(nkcI), where *c* indicates the number of iterations and *I* indicates the dimension of data. Since FCM needs to process fuzzy membership, the calculation process is relatively complicated and usually requires multiple iterations to converge, so the computational time is higher. The efficiency of SC-based SACM is attributed to the fact that the model can be split and coordinated among multiple computing devices, and its streamlined clustering process, which reduces computational overhead while maintaining higher accuracy. This efficiency makes SC-based SACM particularly suitable for real-time applications, where rapid processing is crucial. The significant reduction in runtime not only demonstrates the computational superiority of SC-based SACM but also its potential for large-scale deployment in practical scenarios.

To sum up, SACM integrates adaptive background removal and cluster number selection using the elbow method, which is specifically tailored for levee piping hazard identification in UAV thermal infrared imagery. Unlike classical methods that cluster the entire image and require a preset cluster number, SACM first eliminates irrelevant background and then adaptively determines the optimal number of clusters based on data characteristics. This reduces the influence of noise and background interference, leading to more accurate and robust extraction of piping hazard regions. Additionally, SACM’s initialization based on thermal histogram peaks further enhances clustering stability. These domain-specific adaptations enable SACM to outperform general-purpose algorithms, especially in complex and noisy scenarios, as demonstrated in our comparative results.

In this study, a comprehensive set of evaluation metrics—including precision, recall, accuracy, and F1-score—was used to assess the performance of the proposed SACM algorithm and several classical clustering algorithms (KM, GMM, FCM). As shown in Table 3, the SACM algorithm achieved a recall of 0.70 and an F1-score of 0.26, which are significantly higher than those of the benchmark algorithms (recall: 0.06–0.07, F1-score: 0.11–0.12). This demonstrates SACM’s strong ability to identify true hazard regions and minimize missed detections, which is critical in practical levee piping hazard inspection scenarios, where missing a hazard could have severe consequences. Although SACM’s precision (0.16) is lower than that of the other methods, this trade-off is acceptable in safety-critical applications, as a higher recall is generally preferred to ensure that as many true hazards as possible are detected. The high overall accuracy (0.96–0.97) across all methods reflects the class imbalance in the dataset, where non-hazard regions dominate. Therefore, precision, recall, and F1-score provide a more meaningful evaluation than accuracy alone. The main strength of SACM lies in its domain-adaptive design, which effectively handles background noise and subtle thermal anomalies in UAV imagery. Its high recall and F1-score indicate robust practical applicability for hazard identification. However, the relatively low precision suggests that further optimization may be needed to reduce false positives, especially in large-scale deployments. Future work will focus on enhancing precision by incorporating additional contextual or spatial information.

**Implementing Levee Piping Hazard Inspection on the (Com)^2^INet Platform**. First, the TIR images captured by UAVs and transmitted using a multi-path transmission mechanism that adaptively integrates a space-based network, air-based network, and ground-based network to route data to the (Com)^2^INet platform. Furthermore, the network state is monitored by a network observer module installed inside the centralized SDN controller through a northbound API and an in-band network telemetry (INT) collector. Based on the observations, the serverless platform of (Com)^2^INet constructs a SC-based SACM for the arrival service of levee piping hazard inspection, and schedules resources from a heterogeneous infrastructure pool. The obtained scheduling decisions are then translated into network configuration files, guiding optimal network components to host the required SC to accomplish the computing service in a serial or parallel manner. In addition, to ensure continuous data transmission, uninterrupted critical services, and undegraded service quality, dynamic fault-tolerance mechanisms such as intelligent rerouting, dynamic load balancing, and flexible migration of network functions are employed.

## 4. Open Issues

### 4.1. Computing-Oriented Routing Protocol

In the (Com)^2^INet architecture, the essence of traffic scheduling is to coordinate and optimize communication and computation, forward computing tasks to the optimal network components, and achieve efficient use of multi-dimensional resources and end-to-end QoS and QoE guarantees. This requires comprehensive consideration of network status and available computing resource information to make optimal forwarding decisions to support traffic scheduling. In addition, how to effectively balance resources between communication and computation in routing decision making to avoid resource waste or bottlenecks is still a complex optimization problem [31]. Especially in the case of multi-task concurrency, how to design a computing-oriented routing protocol is an important research topic to achieve ubiquitous connectivity and seamless collaboration of heterogeneous network technologies in (Com)^2^INet computing paradigm.

### 4.2. Deterministic Scheduling for Massive Computing Traffic

Services such as emergency communications and the metaverse require not only computing resources that are available at any time, but also deterministic guarantees for latency, packet loss, etc. to satisfy the strict QoS and QoE requirements. For example, in emergency communications scenarios, the real-time transmission of emergency disaster remote sensing and the rescue instructions requires that data transmission has strict delay and jitter constraints to ensure accurate feedback of disaster situation and timely release of instructions. The core challenge is how to achieve efficient, low-latency resource allocation and path selection for massive computing traffic in a dynamic, heterogeneous network environment [32]. Therefore, developing a deterministic scheduling mechanism for massive computing traffic to support advanced computing services effectively is an urgent issue.

### 4.3. Federated-Enabled Privacy Protection

Security and privacy are critical to all computing paradigms. Although cryptographic techniques such as differential privacy and homomorphic encryption have been used to improve system security and privacy, they may reduce the output accuracy and increase computational overhead. Information sharing and collaboration among multiple providers make the (Com)^2^INet paradigm introduce security and privacy concerns. The core issue is how to achieve a balance between privacy protection and model performance in distributed data collaboration, and how to reduce overhead of computation and communication while ensuring privacy remains an open issue and deserves further study. Federated-enabled mechanism only shares model parameters instead of raw data [33], which is an important research topic for privacy protection and reducing data transmission costs.

## 5. Conclusions

In this work, we present (Com)^2^INet, a novel communication and computation integrated network architecture, to address the challenges of coordinating multi-dimensional heterogeneous resources for computationally intensive and time-sensitive tasks. By integrating sensing, transmission, and computation into a unified framework, (Com)^2^INet enables efficient, intelligent, and scalable collaborative computing across end-edge-cloud, space–air–ground, and multi-data-center domains. Additionally, to demonstrate its applicability, we present a case study of levee piping hazard inspection via remote sensing on the (Com)^2^INet platform. Experimental results show that the proposed SC-based SACM approach achieved superior accuracy with an average mIoU of 0.1469, significantly outperforming traditional methods (KM: 0.0423, GMM: 0.0269, FCM: 0.0303). Furthermore, the approach’s distributed execution reduced computational latency to 0.2288 s per frame, outperforming monolithic benchmarks (KM: 6.7628 s, GMM: 96.3845 s, FCM: 209.3182 s), demonstrating its suitability for real-time emergency scenarios. For future work, we will investigate an adaptive levee piping hazard inspection mechanism based on federated reinforcement learning, enabling (Com)^2^INet to enable data-sensitive computing services from a long-term system performance perspective. Moreover, large-scale simulations and real-world tests will be conducted to verify the performance of the designed algorithm in high-concurrency scenarios.

## Figures and Tables

**Table 1:** Accuracy of target extraction (mIoU).

Piping Number	KM	GMM	FCM	SACM
1	0.0015	0.0266	0.0000	0.4694
2	0.0173	0.0078	0.0019	0.0198
3	0.0157	0.0013	0.0148	0.1094
4	0.0546	0.0600	0.0604	0.1662
5	0.1016	0.0019	0.0908	0.0741
6	0.0712	0.0788	0.0747	0.0755
7	0.0213	0.0182	0.0000	0.0165
8	0.0550	0.0208	0.0000	0.2441
Mean	0.0423	0.0269	0.0303	0.1469

**Table 2:** Computational time (s).

Piping Number	KM	GMM	FCM	SACM
1	6.9079	99.7843	227.2507	0.6001
2	6.21663	93.2404	214.3872	0.1907
3	5.6533	83.7551	217.9142	0.2122
4	6.6272	101.6670	203.7636	0.1717
5	6.3952	99.0716	201.9092	0.1611
6	7.3378	104.0337	207.2638	0.1708
7	6.5378	89.0131	204.2211	0.1601
8	8.4267	100.5110	197.8356	0.1638
Mean	6.7628	96.3845	209.3182	0.2288

**Table 3:** Metrics.

Metrics	KM	GMM	FCM	SACM
precision	0.33	0.38	0.33	0.16
recall	0.06	0.07	0.06	0.7
accuracy	0.97	0.96	0.97	0.96
F1_score	0.11	0.12	0.11	0.26

## Data Availability

The data presented in this study are available on request from the corresponding author due to confidentiality requirements related to levee emergency situations, and further research on the data will be conducted in the future.

## Abbreviations

The following abbreviations are used in this manuscript:

(Com)^2^INet	Communication and computation integrated network
SDN	Software-defined network
NFV	Network function virtualization
QoS	Quality of service
QoE	Quality of experience
AI	Artificial intelligence
VNF	Virtual network function
SC	Service chain
UAV	Unmanned aerial vehicle
SACM	Stepwise adaptive clustering method
TIR	Thermal infrared
KM	k-Medoids
FCM	Fuzzy C-Means
GMM	Gaussian Mixture Model
mIoU	Mean Intersection over Union
NEDT	Noise Equivalent Temperature Difference
